# Meta-Analytic Review of Coronary Angiography in Peri-Procedural Myocardial Injury and Infarction After Cardiac Surgery

**DOI:** 10.3390/jcm14103407

**Published:** 2025-05-13

**Authors:** Alberto Francesco Cereda, Marco Toselli, Paolo Cimaglia, Antonio Gabriele Franchina, Lorenzo Tua, Matteo Carlà, Gabriele Tumminello, Paolo Aseni, Giuseppe Massimo Sangiorgi, Marco Biolcati, Andrea Spangaro, Matteo Rocchetti, Eleonora Pezzoli, Paolo Vanelli, Stefano Lucreziotti

**Affiliations:** 1 Cardiology Unit, ASST Santi Paolo e Carlo, 20153 Milan, Italy; antonio.franchina@asst-santipaolocarlo.it (A.G.F.); lorenzo.tua@asst-santipaolocarlo.it (L.T.); matteo.carla@asst-santipaolocarlo.it (M.C.); marco.biolcati@asst-santipaolocarlo.it (M.B.); andrea.spangaro1@gmail.com (A.S.); matteo.rocchetti@unimi.it (M.R.); eleonora.pezzoli@unimi.it (E.P.); stefano.lucreziotti@asst-santipaolocarlo.it (S.L.); 2 Maria Cecilia Hospital, GVM Care & Research, 48033 Cotignola, Italy; marco.toselli2@gmail.com; 3 Cardiovascular Institute, Azienda Ospedaliero Universitaria di Ferrara, 44121 Ferrara, Italy; paolocimaglia88@gmail.com; 4 Department of Cardio-Thoracic-Vascular Diseases, Foundation IRCCS Ca’ Granda Ospedale Maggiore Policlinico, 20122 Milan, Italy; tumminellogabriele@gmail.com; 5 Department of Emergency Medicine, ASST Grande Ospedale Metropolitano Niguarda, 20162 Milan, Italy; paoloaseni@gmail.com; 6 Cardiac Cath Lab, Department of Biomedicine and Prevention, University of Rome Tor Vergata, 00133 Rome, Italy; gsangiorgi@gmail.com; 7 Cardiac Surgery Unit, Fondazione IRCCS Ca’ Granda Ospedale Maggiore Policlinico, 20122 Milan, Italy; paolo.vanelli@policlinico.mi.it

**Keywords:** peri-procedural myocardial infarction requiring coronary angiography, cardiac surgery complications, graft failure, percutaneous coronary intervention (PCI), graft failure, surgical mortality and outcomes

## Abstract

**Introduction:** Peri-procedural myocardial infarction (PMI) after cardiac surgery is a significant yet often under-recognised complication, sometimes necessitating urgent coronary angiography (PMI-rCA). This meta-analysis evaluates its prevalence, angiographic findings, management strategies, and associated mortality. **Methods:** A systematic review and meta-analysis were conducted according to PRISMA guidelines. Data from nine studies comprising 104,445 post-cardiac surgery patients were analysed. Among them, those undergoing PMI-rCA were categorised by treatment strategy: conservative management, percutaneous coronary intervention (PCI), or reperform surgery. A network meta-analysis compared mortality risks across these groups, with findings visualised using forest plots, network diagrams, and SUCRA rankings. **Results:** PMI-rCA was performed in 1205 patients (2%). Of these, 34.3% had no significant angiographic abnormalities, 53.7% exhibited graft failure, and 10.4% had native vessel ischemia. Management strategies included conservative treatment (55.5%), PCI (23.5%), and reperforming surgery (21%). Network meta-analysis indicated that conservative management was associated with the lowest mortality risk, followed by PCI, while reperforming surgery had the highest risk. **Discussion:** These findings highlight the complexity of PMI diagnosis and treatment. The high proportion of patients without significant angiographic abnormalities raises concerns about potential overuse of invasive procedures. Meanwhile, PCI appears to be a more favourable interventional strategy than reperforming surgery in terms of mortality outcomes. **Conclusions:** PMI requiring coronary angiography is uncommon but clinically significant, with a 16% mortality rate. A tailored, risk-based approach is essential to optimise management, balancing conservative therapy, PCI, and reperforming surgery based on individual patient profiles.

## 1. Introduction

Invasive coronary angiography is not uncommon after cardiac surgery; it is estimated that 1–5% of patients may require an urgent/emergent coronary angiography in the first 30 days after surgery [1]. The clinical indications are different (graft failure, injury or peri-operative infarction, coronary obstruction after valve surgery, ischemia-triggered arrhythmias) and ICA remains the diagnostic gold standard, especially when intervention is needed in the context of PMI/type 5 infarction. Type 5 myocardial infarction (PMI) following cardiac surgery remains a poorly understood and under-recognised condition despite its prognostic significance. Unlike type 1 (spontaneous MI) or type 2 (supply–demand imbalance MI), PMI lacks a standardised diagnostic and therapeutic approach and is a key secondary outcome in comparisons between surgical and percutaneous revascularisation strategies [2]. The definition of Type 5 myocardial infarction varies among different classification systems used for defining PMI, as each criterion uses different biomarker thresholds (cTn or CK-MB) within 48 h post-procedure. The variability between high-sensitivity troponin assays (hs-cTnT and hs-cTnI) and arbitrarily set reference limits makes uniform classification challenging. Therefore, a revision of the peri-procedural myocardial infarction (PMI) definition has been proposed, incorporating more standardised thresholds, imaging evidence, and greater clinical context [3].

The definitions of myocardial injury, type 5 myocardial infarction, and ischemia are inherently overlapping, with subtle interpretative nuances. While myocardial injury is identified by isolated troponin elevation, type 5 MI requires substantial biomarker increase and definitive ischemic evidence; ischemia itself reflects impaired perfusion, not necessarily necrosis. In peri-operative settings, procedural trauma and hemodynamic variability further obscure these boundaries, introducing bias and complicating outcome assessment in clinical research.

Beyond definitions, PMI presents a complex clinical scenario influenced by both patient-specific factors and procedural complications. Etiologies range from ischemia, plaque rupture, and graft failure to thrombus formation, coronary spasm, or extrinsic compression by a prosthetic valve [4]. Clinical presentation often overlaps with other cardiac conditions, manifesting as chest pain, ECG changes, echocardiographic abnormalities, arrhythmias, or hemodynamic instability. Many patients have complex coronary anatomies, multiple comorbidities (e.g., renal dysfunction, diabetes, heart failure), and delayed biomarker detection due to peri-operative factors [5]. Management remains controversial, balancing conservative and interventional strategies, often in hemodynamically unstable patients requiring inotropes or mechanical support [6]. Coronary angiography is crucial but lacks clear indications, varying by institutional protocols and clinical status. Paradoxically, coronary angiography fails to identify a culprit lesion in one-third of cases but guides revascularisation (PCI or redo surgery) in nearly half of patients. Given its impact on outcomes, PMI demands a multidisciplinary approach integrating serial ECGs, biomarkers, non-invasive imaging (e.g., coronary CT), and advanced circulatory support [7]. The arbitrary definition of PMI, increasingly sensitive cardiac markers with increased risk of false positives, the limitations of instrumental diagnostics in the postoperative context, the lack of studies, and the absence of specific guidelines, make PMI a controversial research area of modern cardiology. The purpose of this meta-analysis is to fill, with all the limitations of our study, this clinically relevant knowledge gap [8].

## 2. Methods

### 2.1. Aims

This study aimed to estimate the prevalence of patients with post-procedural myocardial infarction (PMI) requiring coronary angiography (PMI-rCA) and assess the estimated mortality rate reported in the included observational studies. Additionally, it sought to compare mortality rates based on treatment modality, including conservative management, percutaneous coronary intervention (PCI), or reperforming surgery, and to identify study-level variables associated with PMI prevalence and mortality.

### 2.2. Methodology

The meta-analysis and its preliminary methodology were registered on PROSPERO (ID: 646120).

This study followed the PICO framework as follows:

- Population (P): Patients with PMI.
- Intervention (I): Undergoing coronary angiography.
- Comparator (C): Treatment strategy (conservative vs. interventional/surgical therapy).
- Outcome (O): Mortality.

**Figure A1:**
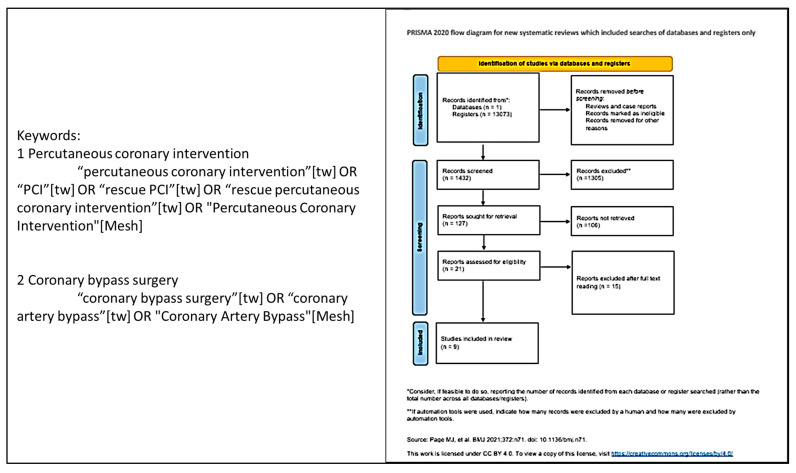
Search strategy and keywords (**left**), meta-analytic flow diagram (**right**).

Observational studies were identified using a predefined key search strategy. A total of 13,084 records were identified through database and registry searches. Following the PRISMA 2020 guidelines, after screening and full-text assessment, 9 studies were included in the review. No randomised controlled trials were identified, as illustrated in Figure A1 (Appendix A).

**Figure A2:**
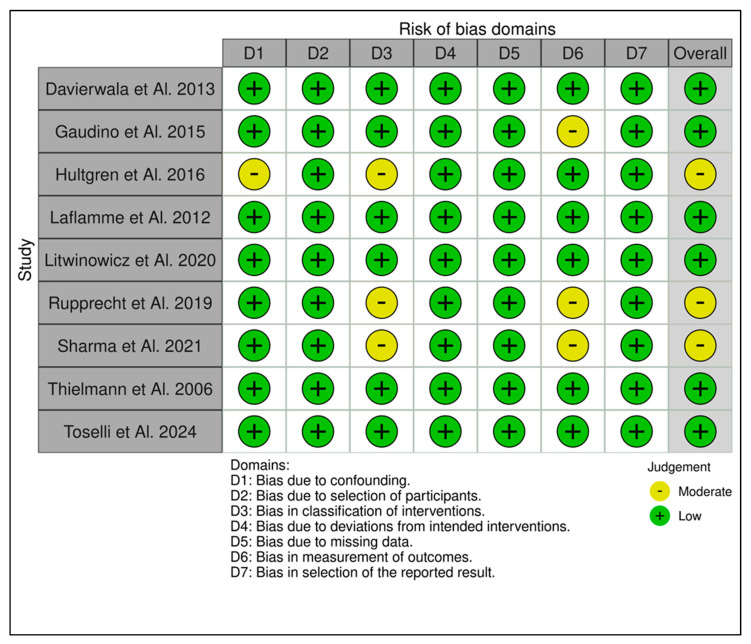
Study bias analysis with ROBINS-E.

The identification, screening, and inclusion process is reported in Appendix A, Table A1. Patients undergoing coronary angiography after cardiac surgery were included; the distinction between injury and myocardial infarction is beyond the scope of this meta-analysis. Two authors (AC and MR) independently performed the study selection process. Any discrepancies were resolved with the assistance of a third reviewer (AF). The literature search was conducted in PubMed and Google Scholar. Data extraction was performed using Excel 365, and only studies that reported mortality stratified by treatment type were included. While most studies exhibited a low overall risk of bias, Hultgren et al. (2016), Rupprecht et al. (2019), and Sharma et al. (2021) showed a higher risk profile, with moderate concerns in multiple domains, particularly regarding intervention classification and outcome measurement. Risk of bias was assessed using the ROBINS-E tool, which evaluates potential biases across seven domains: bias due to confounding, bias in the selection of participants, bias in the classification of exposures, bias due to deviations from intended exposures, bias due to missing data, bias in the measurement of outcomes, and bias in the selection of the reported result (Appendix A, Figure A2). See clinical profile of patients included in Table 1 [9,10,11,12,13,14,15,16,17].

The population analysed in this meta-analysis consisted of patients with peri-operative myocardial infarction after cardiac surgery who underwent coronary angiography. This population, defined by the acronym PMI-rCA (peri-operative myocardial infarction requiring coronary angiography), represents a subset of the broader PMI population. The present meta-analytic analysis specifically refers to the PMI-rCA population.

### 2.3. Statistical Analysis

**Figure 1:**
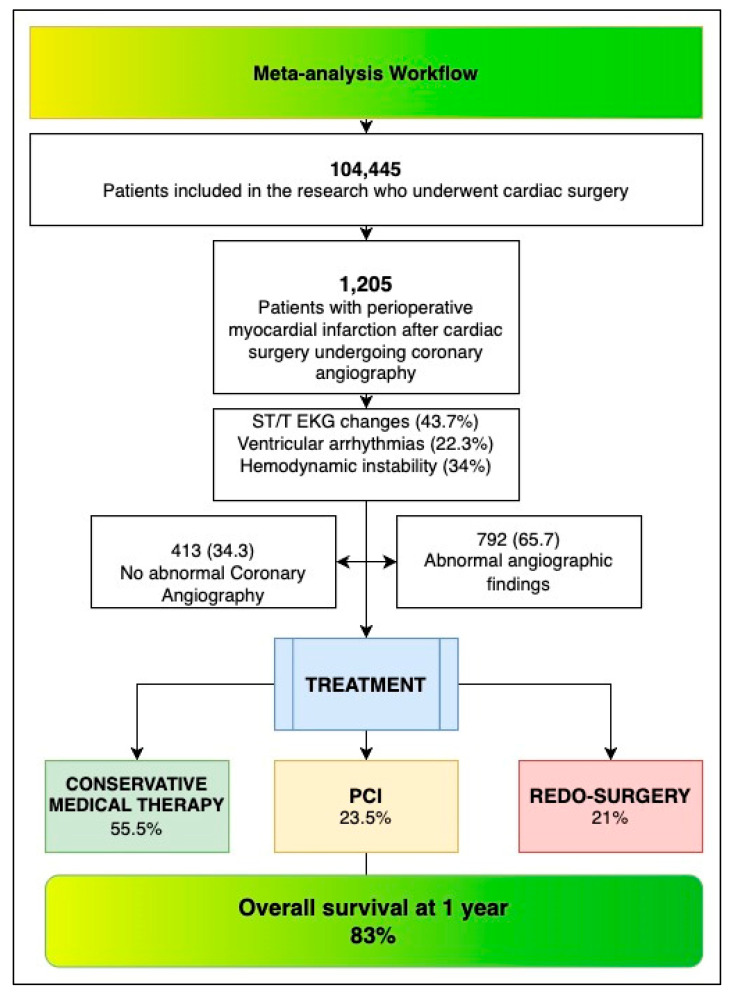
Flowchart of the meta-analysis.

At the study level, demographic data, clinical profiles, and types of cardiac surgery were collected (see Table 1). Coronary angiography findings were categorised as either non-pathological (“Normal Angiography”) or pathological. Patients with abnormal findings were further classified based on their treatment strategy, which included conservative management, percutaneous coronary intervention (PCI), or reperforming surgery. An overview of treatment pathways is provided in Figure 1, while the clinical characteristics of the meta-analytic population are detailed in Table 1 and Table A1 (Appendix A). Mortality was assessed for each subgroup and compared using a network meta-analysis.

Qualitative data were expressed as percentages, while quantitative data were presented as means with standard deviations (SD). The effect size for PMI prevalence and mortality was estimated using the restricted maximum likelihood (REML) model, and heterogeneity was assessed through the I^2^ and the Q test. A random effects model was used for the meta-analysis, with results visualised through forest plots, normalised Q–Q plots for normal distribution assessment, and funnel plots to evaluate publication bias.

Mortality analysis was conducted using a network meta-analysis, where conservative therapy served as the reference group in comparison with PCI and reperforming surgery. Binary mortality outcomes were reported as odds ratios (ORs) within a random-effects model. Results were further illustrated using network plots: the Litmus Rank-O-Gram and the Radial SUCRA plot [18]. Network meta-analysis results were summarized using the Litmus Rank-O-Gram and Radial SUCRA plot, graphical tools designed to improve the readability and interpretation of complex ranking data compared with traditional numerical tables. Meta-regression analyses were conducted for prevalence and mortality in the PMI-rCA population. In the network meta-analysis, the regression curves were all parallel/flat due to the limited power of the analysis at the study level; therefore, they were not reported. Statistical analyses were primarily conducted using STATA, while online tools such as metaHUN were employed for prevalence and mortality estimation, while MetaInsight was used for network meta-analysis visualiation.

## 3. Results

In this meta-analysis, we included 104,445 patients who underwent cardiac surgery across nine studies. Among them, 1205 patients experienced post-procedural myocardial infarction (PMI-rCA) severe enough to require urgent coronary angiography. Of these, 413 patients showed no significant pathological findings, while 792 had abnormalities, and nearly 45% required some interventional or surgical treatment.

**Figure 2:**
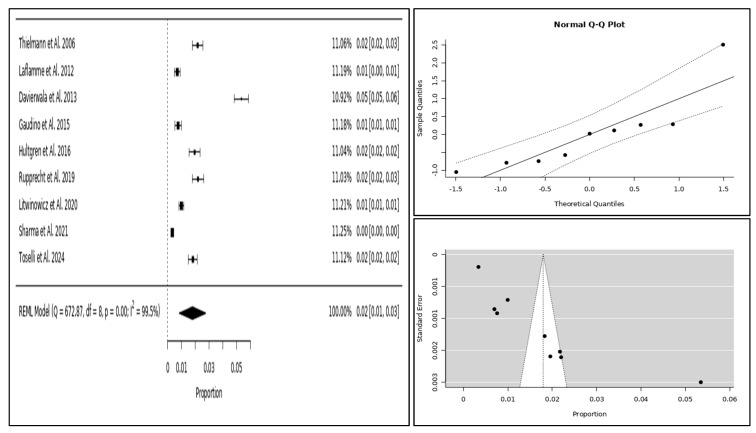
PMI-rCA prevalence displayed with forest plot (**left**), Q–Q plot (**top right**), and funnel plot (**bottom right**).

**Figure 3:**
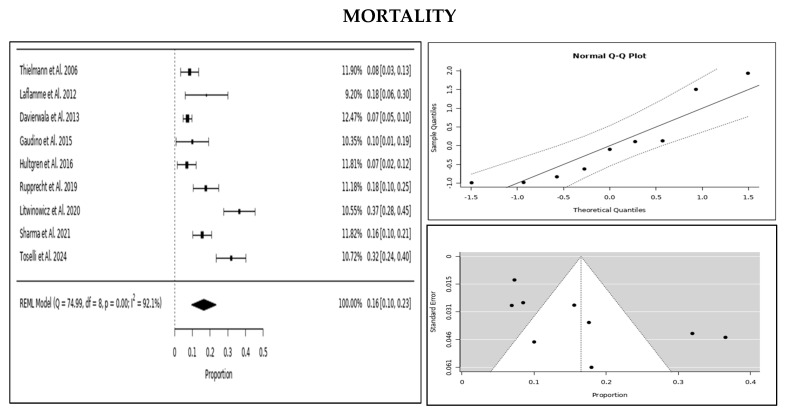
PMI-rCA mortality displayed with forest plot (left), Q–Q plot (**top right**), and funnel plot (**bottom right**).

**Figure 4:**
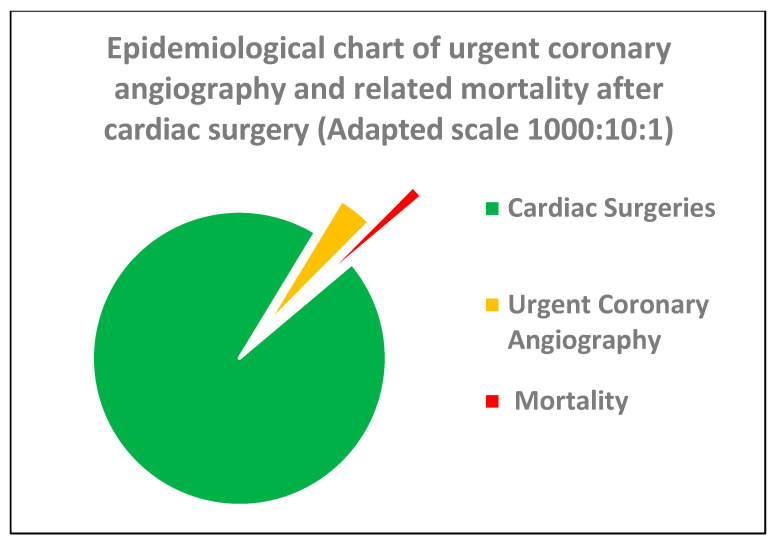
Pie chart showing PMI-rCA prevalence (yellow) and mortality (red).

When looking at overall survival, we found that 83% of these patients were alive at one year. The prevalence of PMI-rCA in this population was estimated at 2%, with a mortality rate of 16% among those requiring coronary angiography. Smaller studies tended to report higher prevalence and mortality rates compared with those with larger patient populations, suggesting a potential overestimation in smaller datasets (Figure 2, Figure 3 and Figure 4).

Looking deeper into the clinical profiles, we found that the average age was 67 years with a female prevalence of 30%; 32.5% of patients were diabetic, and COPD comorbidity was present in 10% of cases. Overall, 36% of patients had a history of previous acute coronary syndrome, 25% of patients had already undergone PCI, and only 5% of patients had already undergone cardiac surgery. It was not possible to analyse the surgical risk in terms of standardised scores (see Table 1).

One-third of patients had a reduced ejection fraction, and half underwent isolated coronary artery bypass grafting (CABG), while almost 7% had isolated valve surgery. A large proportion, 46% of the patients, had combined CABG and valve surgery, making this a significant subgroup. When looking at clinical presentation, ischemic ECG changes were more common than ventricular arrhythmias or instability, and a quarter of patients required circulatory support, primarily an intra-aortic balloon pump (IABP).

Coronary angiography revealed that more than half of the patients (53.7%) had graft failure, while in 10.4%, the issue lay in the native coronary arteries rather than the bypass grafts. Interestingly, one in three patients (34%) had no significant abnormalities on angiography despite their clinical presentation.

**Figure 5:**
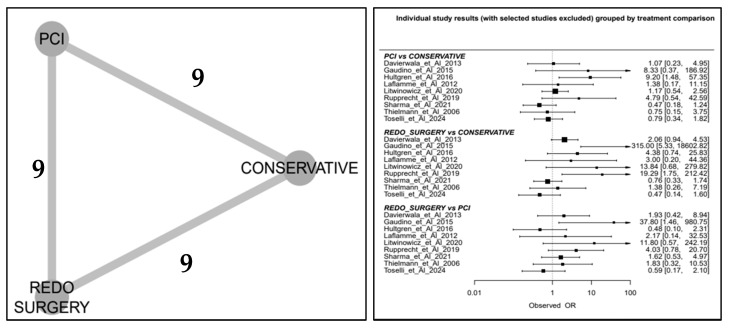
Network plot (**left**) and individual study results compared by treatment (**right**).

**Figure A3:**
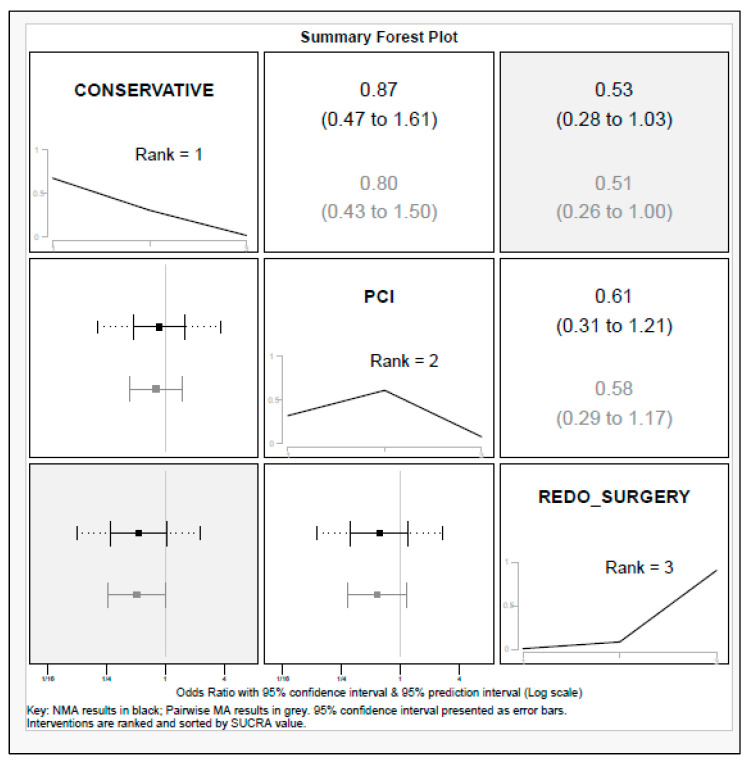
Summary forest plot of NMA.

We observed some notable trends in mortality outcomes when we analysed treatment strategies. Patients who underwent PCI tended to have higher mortality than those managed conservatively, and those who required surgery to be reperformed had the highest mortality of all. However, PCI still appeared to be a better option than reperforming surgery in terms of survival. The network meta-analysis confirmed these trends, showing that conservative management had the lowest mortality risk, followed by PCI, with reperforming surgery carrying the highest risk (Figure 5 and Figure A3 in the Appendix A).

**Figure 6:**
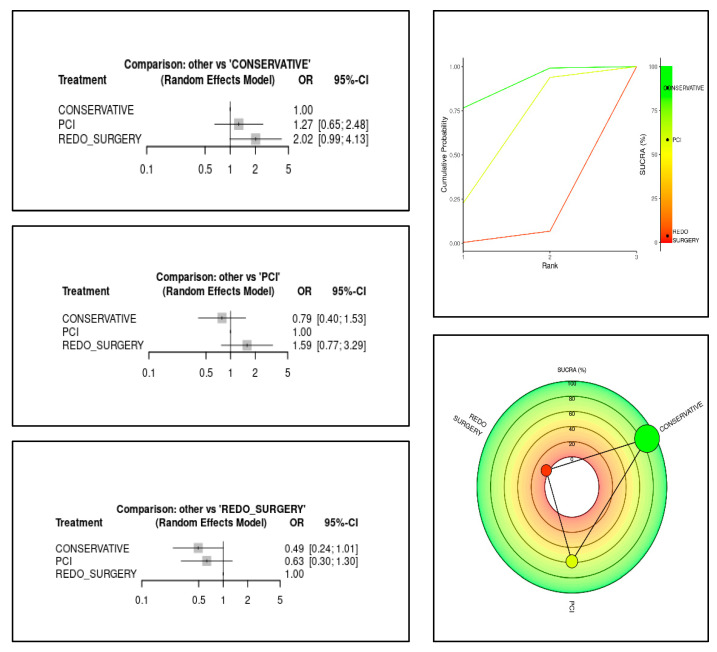
Comparison by treatment (**left)** and graphical representation of the ranking with Litmus Rank-O-Gram (**top right**) and Radial SUCRA (**bottom right**).

Using statistical models, including Litmus Rank-O-Gram and Radial SUCRA plots, we ranked treatment strategies based on cumulative survival probabilities. The results placed conservative treatment as the best-performing option, followed by PCI, and then reperforming surgery (Figure 6). These findings reinforce the idea that in this particular patient population more invasive interventions do not always translate into better outcomes.

**Figure A4:**
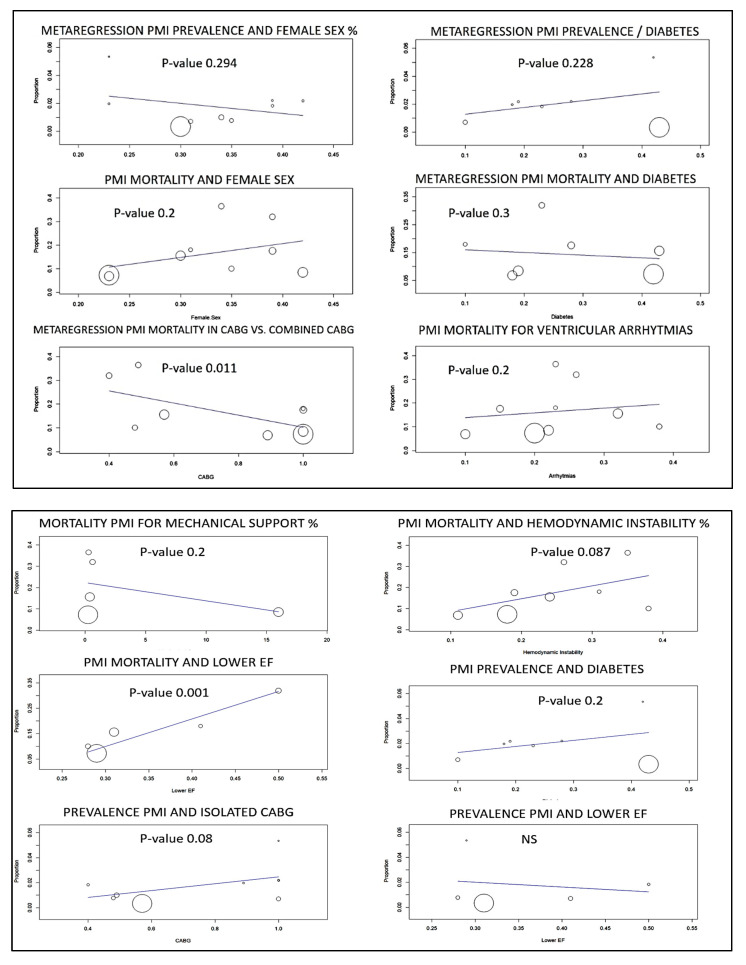
(**top**): meta-regression for PMI-rCA prevalence and mortality. (**bellow**): meta-regression for PMI-rCA prevalence and mortality.

Finally, we explored what factors might predict PMI-rCA prevalence and mortality. Female sex did not seem to influence outcomes. However, CABG surgery was linked to higher PMI prevalence, while combined CABG and valve surgery was associated with increased mortality. Interestingly, having a reduced ejection fraction did not increase the likelihood of developing PMI, but it did correlate with a higher risk of death. The strongest predictor of mortality, however, was hemodynamic instability, which profoundly impacted patient outcomes. Additionally, arrhythmic events and hemodynamic instability often occurred together and were frequently seen in patients who had normal angiographic findings despite their symptoms (Figure A4 in the Appendix A) The meta-regression data within the network meta-analysis were not statistically significant due to the limited statistical power of the analysis. Therefore, it is currently not possible to determine whether clinical presentation variables (such as ST elevation, arrhythmias, or hemodynamic instability) influence the results of the network meta-analysis.

## 4. Discussion

Post-procedural myocardial infarction (PMI) is often under-recognised and misunderstood in clinical practice. The distinction between myocardial injury and infarction remains unclear, but that does not make it any less significant from a prognostic standpoint. PMI is treated as an inevitable side effect of life-saving procedures, but ignoring PMI does not change the fact that even subclinical myocardial injury is increasingly linked to higher mortality and major adverse cardiovascular events (MACE). Rather than being overlooked “collateral damage”, PMI should be seen as an opportunity to better understand myocardial damage and improve outcomes [19].

In the context of PMI, the difference between myocardial injury and infarction can be unclear. Myocardial injury is defined as an isolated rise in cardiac biomarkers above the upper reference limit, without evidence of myocardial ischemia. It may be due to procedural trauma, embolisation, or microvascular dysfunction but does not meet the criteria for infarction. The definition of myocardial infarction (PMI, Type 5 MI) requires biomarker elevation beyond a defined threshold plus at least one additional criterion of myocardial ischemia, such as ECG changes, imaging evidence of new myocardial loss, angiographic documentation of a coronary occlusion, or symptoms consistent with infarction. The distinction can be unclear and coronary angiography can be an invasive diagnostic test [20].

The topic is an “elephant in the room” and there are few comprehensive, retrospective, single-centre studies. The results are sometimes heterogeneous with different interpretative biases

In the study by Toselli et al. (the most recent study included in the meta-analysis), 6505 cardiac surgeries performed between 2016 and 2021 were analysed; they found that 1.83% (119 patients) required urgent coronary angiography during the same hospitalisation. The incidence was higher for post-coronary artery bypass grafting (CABG) at 2.62% compared with 1.35% for non-CABG procedures. Graft failure was detected in 31.1% of cases, and native vessel complications in 36.1% [17].

The study by Thielmann et al. found that urgent percutaneous coronary intervention (PCI) was associated with lower myocardial injury compared to emergency reperformance of surgery and suggests that the early identification and rapid intervention were crucial for improving survival and preserving myocardial function [9].

The study by Litwinowicz et al. analysed 11,537 cardiac surgeries performed over five years, identifying 115 patients (1.19%) who underwent emergency coronary angiography within 24 h post-operation due to hemodynamic instability or suspected myocardial ischemia. Findings from coronary angiography included graft failure (31.3%), native coronary artery occlusion (7.8%), coronary artery embolism (7.0%), coronary artery spasm (4.3%), diffuse atherosclerosis (1.7%), and subclavian artery stenosis (0.9%). No pathological findings were observed in 43.5% of cases. Percutaneous coronary intervention (PCI) was performed in 40% of patients, while 2.6% underwent surgical revascularisation. The overall in-hospital mortality rate was 36.5%, with no significant differences between groups, unlike the Toselli and Thielmann studies [15].

The study by Davierwala et al. (2013) is the most comprehensive to date, with the longest follow-up, and addresses potential interpretative biases in managing peri-operative myocardial ischemia (PMI). Patients were categorised into four groups based on postoperative angiography and treatment: reperform surgery, PCI, conservative treatment, and normal angiogram. Reperform surgery patients had more frequent multiple graft failures, PCI patients often had incomplete revascularisation, and conservative treatment was reserved for clinically stable or high-risk patients. Those with normal angiograms showed no graft-related issues. Preoperative profiles were generally comparable, aside from higher COPD in the PCI group and greater incidence of diabetes in the conservative group. Minimally invasive surgery was more common in PCI patients, and revascularisation occurred more rapidly with PCI than with reperforming surgery. Reperforming surgery was associated with the highest complication and in-hospital mortality rate (10.8%), while PCI had the lowest mortality among patients with graft failure (5.9%). Although conservative treatment showed reduced 5-year survival compared to normal angiogram patients, in-hospital mortality was similar.

In our meta-analysis, we focused specifically on PMI cases severe enough to require invasive coronary angiography after cardiac surgery. The findings reinforce that PMI is not rare, with a 2% prevalence and a 16% mortality rate. To put this into perspective: in a tertiary centre performing 2000 cardiac surgeries per year, that translates to around 40 cases of PMI annually, potentially contributing to a 0.3% post-surgical mortality rate. On a global scale, where 1–2 million cardiac surgeries are performed each year, PMI is far from an insignificant issue (Figure 4).

Interestingly, our meta-regression analysis did not reveal any strong study-level predictors of PMI risk or mortality. Patients with hemodynamic instability and a reduced ejection fraction also had a trend toward higher mortality, though no definitive risk profile for PMI could be established. Even clinical signs such as ST elevation, malignant arrhythmias, or documented graft failure did not correlate with significant differences in mortality. A particularly intriguing finding was that patients who presented with arrhythmias and hemodynamic instability were more likely to have a normal coronary angiography, suggesting a complex interplay between ischemia, electrical instability, and hemodynamic stress rather than just anatomical occlusion [21].

One of the most striking findings of the study was that 34.3% of patients who underwent coronary angiography showed no significant pathology, raising concerns about the potential overuse of invasive angiography in the postoperative setting.

It is important to note that a ‘normal’ coronary angiography in the context of peri-operative myocardial infarction (PMI) after cardiac surgery can be explained by several mechanisms. These include non-atherothrombotic causes of PMI such as oxygen supply–demand imbalance (type 2 MI), transient coronary vasospasm, microembolic events, or myocardial injury related to surgical manipulation or cardiopulmonary bypass. In addition, coronary angiography may fail to detect distal or microvascular obstructions, and the definition of ‘normal’ coronary arteries may include minor, non-critical irregularities. Therefore, a normal angiographic finding does not exclude a peri-operative ischemic event, and this observation highlights the complexity of PMI diagnosis and management in this population.

While coronary angiography remains critical, especially given that 23.5% of patients ultimately required PCI—there is an increasing interest in alternative imaging modalities such as coronary CT angiography (CTA). These non-invasive approaches could help refine diagnostic strategies and potentially reduce unnecessary invasive procedures. However, the role of coronary CTA in the postoperative period remains poorly defined, and further research is needed before it can be incorporated into routine clinical practice [22].

Another key finding was that conservative treatment was the most commonly used strategy (55.5%) and appeared to be associated with lower mortality compared to PCI and redo surgery. Our network meta-analysis was not conclusive enough to establish definitive superiority, but when ranking treatments in terms of survival, conservative therapy consistently came out on top, followed by PCI, with reperforming surgery associated with the highest mortality risk. These results should not be misinterpreted as a reason to avoid intervention—but rather as a call for a more nuanced, patient-specific approach. Instead of defaulting to PCI or reperforming surgery, a more thoughtful risk–benefit discussion is necessary, particularly when handling high-risk, post-cardiac surgery patients [23].

**Figure 7:**
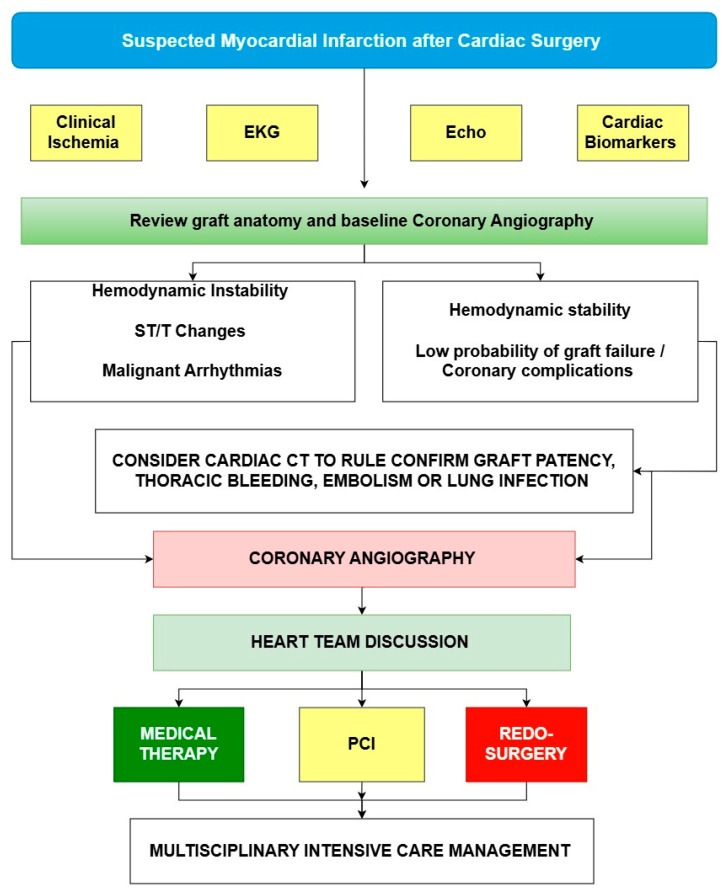
Evidence-informed algorithm for the management of peri-operative myocardial injury-related coronary angiography (PMI-rCA), based on the findings of our systematic review.

One of the biggest challenges in PMI-rCA management is that it often occurs during off-hours, in the context of urgency/emergency. The decision-making process is further complicated by the need for rapid action in critically ill patients in the absence of strong management evidence. To address this, we have developed a management algorithm for PMI cases requiring coronary angiography, providing a structured approach to decision making. (See proposed algorithm in Figure 7).

Clinical decision making in the postoperative setting should begin with a comprehensive clinical interpretation, assessing ischemic symptoms, ECG changes, echocardiographic findings, and biomarkers. A heart team approach is essential, involving interventional cardiologists, cardiac surgeons, intensivists, and clinical cardiologists to guide decisions regarding coronary angiography. In ambiguous cases, coronary CT may be considered when expertise is available to help reduce unnecessary invasive procedures [24]. Before proceeding with angiography, it is crucial to review prior imaging and surgical details, ensuring a thorough understanding of the original surgical strategy. Finally, post-angiography findings should be discussed within the heart team before deciding on PCI or surgery, prioritising optimal patient outcomes rather than solely focusing on treating detected lesions [25].

The therapeutic strategy can also be used stepwise with hybrid solutions, such as PCI, to treat the culprit lesion and then the surgical revascularisation can be proposed again at a later time, or repeat CABG/cardiac surgery and the complete revascularisation of a secondary vessel with PCI [26].

Another notable point from our analysis was the frequent use of an intra-aortic balloon pump (IABP) for hemodynamic support. While IABP was historically a mainstay, its use is now declining in favour of more advanced mechanical circulatory support (MCS) devices like Impella and ECMO. The fact that our study population had such high IABP usage reflects the limitations of historical data, and future research should explore how modern MCS strategies affect PMI outcomes [27,28]. In this meta-analysis, we identified five key clinical takeaways regarding peri-operative myocardial infarction requiring coronary angiography (PMI-rCA) after cardiac surgery. First, although PMI-rCA is relatively uncommon (2% prevalence), it carries a significant 16% mortality risk, underscoring its clinical relevance. Second, nearly one-third of patients undergoing urgent coronary angiography show no critical lesions, raising concerns about potential overuse of invasive diagnostics. Third, conservative management was associated with the lowest mortality rates, suggesting it should be the preferred initial strategy when clinically appropriate. Fourth, reperforming surgical revascularisation was linked to the highest mortality, supporting a cautious, highly selective use of this intervention. Finally, given the absence of clear predictors and the complex clinical presentations, a multidisciplinary, individualised approach remains essential to optimise decision making and outcomes in this high-risk population.

### Limitations of the Research

This meta-analysis is limited by its reliance on observational studies, which, while providing valuable real-world data, lack the control of randomised trials. Nevertheless, the inclusion of multiple studies strengthens the overall findings.

This study is based on observational data, as no randomised controlled trials (RCTs) were available on this topic. PMI itself remains a heterogeneous entity, with definitions still evolving, making standardisation difficult. Additionally, since we specifically analysed PMI cases requiring coronary angiography, there is a deliberate selection bias in this dataset. The time range across the included studies is wide, extending from the study by Thielmann published in 2006 to the most recent by Toselli in 2024; moreover, as all studies were retrospective, temporal variability may have influenced clinical practices and may not fully reflect the contemporary management of peri-operative ischemia.

Analysing the available data on mechanical circulatory support in our study, the relatively low use of ECMO and the higher reliance on IABP, compared with more contemporary cohorts, may be attributed to the evolution of clinical practices and the progressive availability of advanced circulatory support technologies over time.

The interpretation of our study findings must be contextualised in light of a potential time-related bias, encompassing both historical and technological components; the generalisability of our results is inevitably influenced by these biases.

That said, the goal of this study is not to provide absolute answers, but rather to highlight the complexity of PMI and encourage further investigation. Future large-scale registries and multicentre studies will be necessary to provide clearer guidance.

The approach must be tailored to the patient, individualised to their coronary anatomy, the territory of ischemia distribution, and surgical/procedural risk. Furthermore, the therapeutic choice must be shared in a multidisciplinary team without therapeutic prevarications. In the case of non-critical ischemia without arrhythmias, and hemodynamic instability, medical therapy will be chosen. In the case of occlusion of a venous graft, it is perhaps better to resort to PCI rather than surgery. The therapeutic choice can also be sequential and resort to surgery in the case of PCI failure. In the case of cardiogenic shock, the choice of PCI/re-surgery will also depend on the hemodynamic support used, in favour of PCI in the case of IABP/Impella and surgery if ECMO is used in patients with arterial graft failure or residual valvular dysfunction.

Future research perspectives: Although the present meta-analysis helps to fill an important knowledge gap regarding PMI-rCA, many unresolved issues remain. We consider this study a starting point for future prospective research, and it is our intention that this meta-analysis may at least serve as a reference point for the development of a dedicated registry.

## 5. Conclusions

PMI requiring invasive coronary angiography is not a rare occurrence (2%), and its impact on mortality is significant (16%). Despite analysing multiple variables, no clear study-level predictors emerged that could help better phenotype this complication. However, within the limitations of this meta-analysis, we observed a trend favouring conservative therapy over PCI and reperforming surgery in terms of lower mortality risk. These findings should serve as a starting point for further research, rather than a definitive guideline. Ultimately, PMI-rCA management should be multidisciplinary, with decisions tailored to each patient’s risk profile and clinical presentation. The best approach is likely a combination of optimal medical therapy, advanced imaging, and a thoughtful heart team discussion before proceeding with PCI or surgery. With more research and better-defined protocols, we can move towards minimising the impact of PMI-rCA and improving outcomes for post-cardiac surgery patients.

## Figures and Tables

**Table 1:** Patient characteristics, surgery type, urgent CAG indications, support strategies, angiographic findings, management, and outcomes.

Demographic and Clinical Profile	
All patients undergoing cardiac surgery, N	104,445
Number of patients with peri-procedural MI, N	1205
Mean age, years ± SD	67 ± 2
Female sex, n/N(%)	365 (30.3)
Hypertension, n/N(%)	928 (77)
Dyslipidemia, n/N(%)	786 (65)
Diabetes, n/N(%)	341 (32.5)
Hystory of Smoking, n/N(%)	254 (39)
COPD, n/N(%)	91 (10)
Prior ACS, n/N(%)	157 (36)
Prior PCI, n/N(%)	185 (24.3)
Prior Heart Surgery, n/N(%)	33 (4.9)
EF < 55%, n/N(%)	258 (33)
**Type of Surgery**	
Isolated CABG Surgery, n/N(%)	568 (47.1)
Isolated Valve Surgery, n/N(%)	83 (6.9)
Combine Surgery, n/N (%)	554 (46)
Cross-Clamping Time, min ± SD	63 ± 38
**Clinical Indication for Cag**	
ST/T EKG Abnormalities, n/N(%)	526 (43.7)
Ventricular Arrhythmias, n/N(%)	269 (22.3)
Hemodinamic Instability, n/N(%)	410 (34)
**Mechanical Circulatory Support**	
Mechanical Support, n/N(%)	304 (25.2)
IABP insertion, n/N(%)	248 (20.5)
CPR/Resuscitation, n/n (%)	160 (13.2)
ECMO, n/N(%)	46 (3.8)
**Cag Findings**	
Graft Failure, n/N(%)	647 (53.7)
Native Vessel Ischemia, n/N(%)	125 (10.4)
Non-Critical Angiography Findings, n/N(%)	413 (34.2)
Spasm, n/N(%)	20 (1.7)
**Treatment**	
Conservative Treatment, n/N(%)	669 (55.5)
PTCA, n/N(%)	284 (23.5)
Redo Surgery, n/N(%)	252 (21)
**Outcomes**	
30 d mortality in conservative treatment, n/N(%)	92 (14.9)
30 d mortality in PTCA, n/N(%)	55 (19.3)
30 d mortality in Redo Surgery, n/N(%)	46 (18.3)
30 d mortality, n/N(%)	193 (16)
One year mortality, n/N(%)	204 (16.9)

## Data Availability

The dataset used in this meta-analysis was derived from the results of nine published studies. Requests for access to the extracted and analysed data can be directed to the corresponding author.

## Abbreviations

The following abbreviations are used in this manuscript:

CABG	Coronary Artery Bypass Grafting
cTn	Cardiac Troponin
CTA	Coronary Computed Tomography Angiography
ECG (EKG)	Electrocardiogram
ECMO	Extracorporeal Membrane Oxygenation
IABP	Intra-Aortic Balloon Pump
MACE	Major Adverse Cardiovascular Events
MCS	Mechanical Circulatory Support
MI	Myocardial Infarction
PCI	Percutaneous Coronary Intervention
PMI	Peri-procedural Myocardial Infarction
PMI-rCA	Peri-procedural Myocardial Infarction requiring Coronary Angiography

## Appendix A

**Table A1:** Variables reported for each study included in the meta-analysis.

Variables	Davierwala et al.	Gaudino et al.	Laflamme et al.	Litwinowicz et al.	Sharma et al.	Toselli et al.	Thielmann et al.	Rupprecht et al.	Hultgren et al.
Total patients, N	7461	5275	5598	11,537	53,287	6505	5427	4909	4446
Patient with PMI, n/N	399	40	39	115	180	119	118	108	87
Not survided (with evidence of PMI), n/N	29	4	7	42	28	38	10	19	6
Age, years	68	71	66	66	66	69	66	66	67
Female Sex, %	0.23	0.35	0.31	0.34	0.30	0.39	0.42	0.39	0.22
Hypertension, %	0.91	0.90	0.56	0.80	0.81	0.67	0.36	0.79	0.69
Dyslipidemia, %	0.76	0.58	0.49	0.67	0.73	0.60	0.38	0.64	0.56
Diabetes, %	0.42	-	0.10	-	0.43	0.23	0.19	0.28	0.17
Smoke, %	-	-	0.33	-	0.54	0.50	0.28	0.37	0.11
COPD, %	0.06	0.55	0.13	-	-	0.10	0.08	0.11	0.06
Prior ACS, %	-	0.13	0.44	0.60	-	0.22	0.34	-	-
Prior PTCA, %	0.25	-	0.15	0.31	-	0.16	-	-	0.28
Lower EF, %	0.29	0.28	0.41	-	0.31	0.50	-	-	-
Triple vessel disease, %	0.73	0.33	0.10	-	-	-	0.43	0.91	0.59
Left main, %	0.09	0.15	0.36	-	-	-	-	0.48	0.43
Isolated CABG, %	0.00	0.48	1.00	0.49	0.57	0.40	1.00	1.00	0.89
Valve Surgery, %	-	0.38	-	0.08	0.07	0.39	-	-	-
ST/T abnormalities, %	0.50	0.63	0.59	0.25	0.29	0.39	0.09	0.67	0.78
Ventricular arrhytmias, %	0.20	0.38	0.23	0.23	0.32	0.26	0.22	0.15	0.10
Graft failure (total), %	0.64	0.43	0.82	0.31	0.45	0.31	0.57	0.65	0.60
Only medical therapy, %	0.59	0.55	0.51	0.57	0.54	0.46	0.66	0.43	0.67
Operative revascularization, %	0.41	0.45	0.49	0.43	0.46	0.38	0.30	0.57	0.45
Redo Surgery, %	0.33	0.08	0.10	0.03	0.24	0.16	0.21	0.09	0.29
PTCA, %	0.09	0.38	0.38	0.40	0.21	0.38	0.13	0.48	0.16
Mechanic support, %	0.25	-	-	0.28	0.40	0.63	0.22	-	-
IABP insertion, %	0.22	-	-	0.28	0.29	0.49	0.16	-	-
ECMO, %	0.03	-	-	-	-	0.14	0.12	-	0.02
Mortality 1 year, %	0.10	0.15	0.13	0.37	0.23	0.34	0.12	0.18	0.11

## References

[1] Gaudino M., Dangas G.D., Angiolillo D.J., Brodt J., Chikwe J., DeAnda A., Hameed I., Rodgers M.L., Sandner S., Sun L.Y. (2023). Considerations on the Management of Acute Postoperative Ischemia After Cardiac Surgery: A Scientific Statement from the American Heart Association. Circulation.

[2] Jørgensen P.H., Nybo M., Jensen M.K., Mortensen P.E., Poulsen T.S., Diederichsen A.C., Mickley H. (2014). Optimal cut-off value for cardiac troponin I in ruling out Type 5 myocardial infarction. Interact. Cardiovasc. Thorac. Surg..

[3] Gaudino M., Jaffe A.S., Milojevic M., Sandoval Y., Devereaux P.J., Thygesen K., Myers P.O., Kluin J. (2024). Great debate: Myocardial infarction after cardiac surgery must be redefined. Eur. Heart J..

[4] Gaudino M., Flather M., Capodanno D., Milojevic M., Bhatt D.L., Biondi Zoccai G., Boden W.E., Devereaux P.J., Doenst T., Farkouh M. (2024). European Association of Cardio-Thoracic Surgery (EACTS) expert consensus statement on perioperative myocardial infarction after cardiac surgery. Eur. J. Cardiothorac. Surg..

[5] Nanni S., Garofalo M., Schinzari M., Nardi E., Semprini F., Battistini P., Barberini F., Foà A., Baiocchi M., Castelli A. (2022). Prognostic value of high-sensitivity cardiac troponin I early after coronary artery bypass graft surgery. J. Cardiothorac. Surg..

[6] Steuer J., Hörte L.-G., Lindahl B., Ståhle E. (2002). Impact of perioperative myocardial injury on early and long-term outcome after coronary artery bypass grafting. Eur. Heart J..

[7] Rasmussen C., Thiis J.J., Clemmensen P., Efsen F., Arendrup H.C., Saunamäki K., Madsen J.K., Pettersson G. (1997). Significance and management of early graft failure after coronary artery bypass grafting: Feasibility and results of acute angiography and re-re-vascularization. Eur. J. Cardiothorac. Surg..

[8] Heuts S., Gollmann-Tepeköylü C., Denessen E.J.S., Olsthoorn J.R., Romeo J.L.R., Maessen J.G., van ‘t Hof A.W.J., Bekers O., Hammarsten O., Pölzl L. (2023). Cardiac troponin release following coronary artery bypass grafting: Mechanisms and clinical implications. Eur. Heart J..

[9] Thielmann M., Massoudy P., Jaeger B.R., Neuhäuser M., Marggraf G., Sack S., Erbel R., Jakob H. (2006). Emergency re-revascularization with percutaneous coronary intervention, reoperation, or conservative treatment in patients with acute perioperative graft failure following coronary artery bypass surgery. Eur. J. Cardiothorac. Surg..

[10] Laflamme M., DeMey N., Bouchard D., Carrier M., Demers P., Pellerin M., Couture P., Perrault L.P. (2012). Management of early postoperative coronary artery bypass graft failure. Interact. Cardiovasc. Thorac. Surg..

[11] Davierwala P.M., Verevkin A., Leontyev S., Misfeld M., Borger M.A., Mohr F.W. (2013). Impact of expeditious management of perioperative myocardial ischemia in patients undergoing isolated coronary artery bypass surgery. Circulation.

[12] Gaudino M., Nesta M., Burzotta F., Trani C., Coluccia V., Crea F., Massetti M. (2015). Results of emergency postoperative re-angiography after cardiac surgery procedures. Ann. Thorac. Surg..

[13] Hultgren K., Andreasson A., Axelsson T.A., Albertsson P., Lepore V., Jeppsson A. (2016). Acute coronary angiography after coronary artery bypass grafting. Scand. Cardiovasc. J..

[14] Rupprecht L., Schmid C., Debl K., Lunz D., Flörchinger B., Keyser A. (2019). Impact of coronary angiography early after CABG for suspected postoperative myocardial ischemia. J. Cardiothorac. Surg..

[15] Litwinowicz R., Filip G., Bryndza M., Bartus M., Sadowski J., Kapelak B., Mazur P., Vuddanda V., Lakkireddy D., Bartus K. (2020). Outcomes of emergency coronary angiography after cardiac surgery. Eur. J. Prev. Cardiol..

[16] Sharma V., Chen K., Alansari S.A.R., Verma B., Soltesz E.G., Johnston D.R., Tong M.Z., Roselli E.E., Wierup P., Pettersson G.B. (2021). Outcomes of Early Coronary Angiography or Revascularization After Cardiac Surgery. Ann. Thorac. Surg..

[17] Toselli M., Cimaglia P., Cereda A., Fabbri G., Latta F., Giovannini D., Galli M., Calvi S., Nerla R., Castriota F. (2025). Urgent Coronary Angiography Following Cardiac Surgery: Insights from a High-Volume Cardiac Surgery Center. Catheter. Cardiovasc. Interv..

[18] Nevill C.R., Cooper N.J., Sutton A.J. (2023). A multifaceted graphical display, including treatment ranking, was developed to aid interpretation of network meta-analysis. J. Clin. Epidemiol..

[19] Spagnolo M., Occhipinti G., Laudani C., Greco A., Capodanno D. (2024). Periprocedural myocardial infarction and injury. Eur. Heart J. Acute Cardiovasc. Care.

[20] Pölzl L., Thielmann M., Cymorek S., Nägele F., Hirsch J., Graber M., Sappler N., Eder J., Staggl S., Theurl F. (2022). Impact of myocardial injury after coronary artery bypass grafting on long-term prognosis. Eur. Heart J..

[21] Hara H., Serruys P.W., Takahashi K., Kawashima H., Ono M., Gao C., Wang R., Mohr F.W., Holmes D.R., Davierwala P.M. (2020). SYNTAXExtended Survival Investigators Impact of Peri-Procedural Myocardial Infarction on Outcomes After Revascularization. J. Am. Coll. Cardiol..

[22] Di Lazzaro D., Crusco F. (2017). CT angio for the evaluation of graft patency. J. Thorac. Dis..

[23] Trivedi D.P., Chigarapalli S.R., Gangahar D.M., Machiraju V.R. (2021). The impact of advances in percutaneous catheter interventions on redo cardiac surgery. Indian. J. Thorac. Cardiovasc. Surg..

[24] Lee D.W., Cavender M.A. (2019). Periprocedural Myocardial Infarction in Contemporary Practice. Interv. Cardiol. Clin..

[25] Hanna E.B., Hennebry T.A. (2010). Periprocedural myocardial infarction: Review and classification. Clin. Cardiol..

[26] Felekos I., Theodoropoulos K.C., Mullen L. (2020). Acute Circumflex Occlusion After a Successful Mitral Valve Repair. Cardiovasc. Revascularization Med..

[27] Imamura T., Oshima A., Narang N., Onoda H., Tanaka S., Ushijima R., Sobajima M., Fukuda N., Ueno H., Kinugawa K. (2022). Clinical implications of troponin-T elevations following TAVR: Troponin Increase Following TAVR. J. Cardiol..

[28] Sabra M.J., Andrews W.G., Crandall M.L., Pirris J.P. (2020). The postoperative use of Impella as a ventricular assist device in high-risk patients undergoing coronary artery bypass surgery: A case series and comparison. J. Card. Surg..

